# Peer Review of “Representing Physician Suicide Claims as Nanopublications: Proof-of-Concept Study Creating Claim Networks”

**DOI:** 10.2196/40299

**Published:** 2022-07-01

**Keywords:** physician suicide, suicide, suicide prevention, physician well-being, physician mental health, nanopublication, physician, doctor, mental health, semantic publishing, bibliometrics, claim network, information distortion, misinformation

*This is a peer-review report submitted for the paper “Representing Physician Suicide Claims as Nanopublications: Proof-of-Concept Study Creating Claim Networks.”*

## Round 1 Review

### General Comments

This paper [1] integrates these various claims, enables the verification of nonauthoritative assertions, and makes informed statements in the advocacy of physician suicide prevention, thereby better equipping researchers and advancing evidence-based knowledge.
